# Vitamin D Status in a Large, Ethnically Diverse Patient Population Living in South East London at the Onset of the COVID-19 Pandemic: A Cross-Sectional Study Including a SARS-CoV-2 Positive Patient Subset

**DOI:** 10.3390/nu17172861

**Published:** 2025-09-04

**Authors:** Agata Sobczyńska-Malefora, Aleksander Sulkowski, Laurence Harbige, David Steed, Dominic Jon Harrington

**Affiliations:** 1 The Nutristasis Unit, Synnovis, St. Thomas’ Hospital, London SE1 7EH, UK; 2 Faculty of Life Sciences and Medicine, King’s College London, London SE1 1UL, UK; 3 Centre for Health and Life Sciences Research, London Metropolitan University, London N7 8DB, UK; 4 Faculty of Infectious and Tropical Diseases, London School of Hygiene and Tropical Medicine, London WC1E 7HT, UK; 5 Application Management Services, Viapath, St. Thomas’ Hospital, London SE1 7EH, UK; 6 School of Medicine, University of Surrey, Guildford GU2 7XH, UK

**Keywords:** vitamin D, SARS-CoV-2, COVID-19, ethnicity, age, infection, inflammation

## Abstract

**Background/Objectives:** Vitamin D is involved in immune regulation, and deficiency may increase susceptibility to SARS-CoV-2 infection. This study assessed vitamin D status and examined associations between serum 25-hydroxyvitamin D (25(OH)D) concentrations and demographic, anthropometric, and clinical factors, including SARS-CoV-2 infection, in a diverse urban UK patient population. **Methods:** We analysed 25(OH)D concentrations in 17,619 pre-COVID-19 vaccine patients (62% female) whose samples were routinely processed between January and June 2020 at St Thomas’ Hospital, London, UK. SARS-CoV-2 RNA/IgG test results (March 2020–January 2021) were linked to these records. Associations were examined with age, BMI, sex, ethnicity, and laboratory data. Vitamin D deficiency was defined as 25(OH)D <25 nmol/L, and insufficiency as 25–50 nmol/L. **Results:** Vitamin D deficiency was observed in 25% of Black, 21% of Asian, and 17% of White patients; insufficiency was found in 36%, 34%, and 33%, respectively. Serum 25(OH)D concentrations differed by sex in Black and White patients but not in Asian patients. A total of 485 patients (2.8%) were SARS-CoV-2 positive, with a median 25(OH)D concentration of 42 nmol/L (IQR 25–66); 24.1% were deficient and 36.7% insufficient (60.8% total). Among deficient individuals, 38% were White (median age 67.5 years) and 35% Black (median age 52.0 years). Age and BMI were the most significant contributors to infection in White and Black patients, respectively. **Conclusions:** Vitamin D deficiency and insufficiency were common across all ethnic groups and associated with SARS-CoV-2 infection. Deficiency was most prevalent among Black patients. Vitamin D status should be monitored in patient populations, and deficiencies addressed to ensure adequacy of this nutrient for immune system regulation and possibly the reduction in respiratory infection risk, including COVID-19.

## 1. Introduction

Severe outcomes from SARS-CoV-2 infection resulting in COVID-19 are associated with risk factors such as advanced age, high BMI, male sex, certain comorbidities, and ethnicity, particularly among individuals from Black, Asian, and Minority Ethnic (BAME) backgrounds [1]. In England, Wales, and Northern Ireland, early data indicated that 34% of critically ill COVID-19 patients—defined here as those requiring admission to intensive care units (ICU)—came from BAME backgrounds [2], despite this group comprising approximately 18% of the total population [3].

Vitamin D deficiency, defined as serum 25-hydroxyvitamin D [25(OH)D] concentration below 25 nmol/L, and insufficiency (25–50 nmol/L) are more prevalent in BAME populations [4,5], partly due to darker skin pigmentation reducing the efficiency of dermal synthesis from sunlight. In the UK, this is compounded by the seasonal lack of UVB radiation from October to March. Although stores can accumulate through sunlight exposure from April to September, UK public health guidelines recommend vitamin D supplementation during the winter months, especially for high-risk groups [6,7]. Data from the UK Biobank revealed that over 50% of individuals identifying as Asian and nearly 40% of those of Black African descent were vitamin D deficient in the winter and spring months. In contrast, deficiency rates among White individuals were significantly lower across all seasons [8].

Vitamin D plays a critical role in immune regulation, as summarised in a systematic review [9], which highlights its influence on both innate and adaptive immune responses. It acts via the vitamin D receptor (VDR), regulating the expression of over 1000 genes [10,11,12], including those involved in the immune response and inflammation suppression [13,14,15]. Vitamin D downregulates pro-inflammatory cytokines such as tumour necrosis factor alpha (TNF-ɑ), interleukin (IL)-6, and interferon (IFN)-γ [16,17,18], upregulates anti-inflammatory mediators like IL-10 and transforming growth factor beta (TGF-β) [11,19,20,21], promotes differentiation of regulatory T cells [22,23], and reduces immune cell activation [22,24]. It also enhances the expression of anti-microbial peptides [25,26,27] and promotes the expression of the soluble angiotensin-converting enzyme 2 (ACE2), which plays a role in suppressing inflammation and preventing binding of SARS-CoV-2 to cellular receptors [9,28,29]. These functions are particularly relevant in the context of COVID-19, where dysregulated immune responses and cytokine storms have been associated with severe disease and increased mortality [30].

Despite the known immunological functions of vitamin D and the disproportionate impact of COVID-19 on BAME communities, limited data exist on how vitamin D status varies by demographic group during the pandemic, and whether it is associated with SARS-CoV-2 infection or inflammatory markers in real-world settings. A better understanding of these associations may provide insights into potential mechanisms underlying the increased vulnerability observed in certain populations.

Therefore, this study was conducted to assess vitamin D status in patients from South East London during the early stages of the COVID-19 pandemic—and importantly—prior to the availability of vaccines and before widespread interventions could influence population immunity. This unique temporal context offers a rare window into the unmitigated host–pathogen interactions in SARS-CoV-2 infection, unconfounded by vaccine-induced immunity. We aimed to evaluate the relationship between serum 25(OH)D concentrations and demographic factors such as age, BMI, sex, and ethnicity. Additionally, we investigated associations between vitamin D status, SARS-CoV-2 positivity, and circulating pro-inflammatory markers, e.g., cytokine concentrations, to explore whether vitamin D inadequacy may have contributed to differential immune responses.

## 2. Methods

### 2.1. Study Population

A total of 17,619 serum 25(OH)D laboratory test results were retrospectively obtained from the Nutristasis Unit at St Thomas’ Hospital, London, UK, covering the period from January to June 2020. These results were linked to corresponding demographic and anthropometric data, where available, including age, self-identified gender, ethnicity (*n* = 12,242), and height and weight (*n* = 4106). Additional biochemical and haematological data were extracted for individuals who had relevant tests conducted within two weeks of the vitamin D measurement. These included adjusted calcium (*n* = 11,655), total calcium (*n* = 11,708), C-reactive protein (CRP; *n* = 4931), full blood count (FBC; *n* = 15,183), ferritin (*n* = 9581), folate (*n* = 8661), vitamin B12 (*n* = 5122), liver profile (*n* = 12,089), magnesium (*n* = 1920), parathyroid hormone (PTH; *n* = 2213), phosphate (*n* = 11,469), and renal profile (*n* = 14,021). All data were extracted from the Pathnet and Electronic Patient Records systems at St Thomas’ Hospital.

SARS-CoV-2 infection status was available for 485 individuals and was matched anonymously to the vitamin D dataset. Infection was confirmed via detection of either viral RNA or SARS-CoV-2-specific IgG antibodies. These data covered the period from March 2020 to January 2021. In addition, a subset of 45 SARS-CoV-2-positive serum samples, obtained from consecutive patients referred for cytokine analysis, was used to evaluate associations between vitamin D status and inflammatory markers.

### 2.2. Analytical Methods

Serum 25-hydroxyvitamin D [25(OH)D] concentrations were measured using the Abbott Architect 2000 series immunoassay system (Abbott Diagnostics, Lake Forest, IL, USA), which quantifies the sum of 25(OH)D_2_ and 25(OH)D_3_. For samples with concentrations exceeding 150 nmol/L, confirmatory testing was performed using liquid chromatography–tandem mass spectrometry (LC-MS/MS). Both methods are enrolled in the External Quality Assessment Scheme (DEQAS, London, UK) and demonstrate within- and between-assay coefficients of variation of less than 10%. Vitamin D status was categorised as deficient (<25 nmol/L), insufficient (25–50 nmol/L), or replete (>50 nmol/L). Routine biochemistry assays were processed using Roche instrumentation following standard protocols.

SARS-CoV-2 positivity was confirmed via detection of viral RNA or IgG antibodies. For symptomatic individuals and confirmed cases, viral RNA detection was performed using the AusDiagnostics multiplex-tandem polymerase chain reaction (MT-PCR) assay (Mascot, Australia) [31]. For screening of asymptomatic patients, testing was conducted using the Aptima™ SARS-CoV-2 transcription-mediated amplification (TMA) assay on the Panther system (Hologic, Marlborough, MA, USA) [32]. Both assays were used for the qualitative detection of SARS-CoV-2 RNA in nasopharyngeal and oropharyngeal swab specimens, according to manufacturers’ instructions. Serological testing for IgG antibodies to SARS-CoV-2 was initially performed using the Abbott Architect assay (Abbott Diagnostics) targeting nucleocapsid protein and, from November 2020 onwards, the DiaSorin Liaison XL (Saluggia, Italy) assay targeting spike protein. A high level of concordance between these assays has previously been demonstrated [33].

A cytokine panel comprising IL-1β, IL-6, IL-8, and TNF-ɑ was analysed. The selection of these cytokines, along with the rationale for their inclusion, sample collection protocols, and assay validation procedures, has been detailed previously [34]. Cytokine concentrations were measured using the Simple Plex™ Ella immunoassay platform (ProteinSimple, Bio-Techne, Oxford, UK). The assay demonstrated a coefficient of variation of less than 10% for all analytes measured.

### 2.3. Statistical Analyses

Descriptive statistics [frequencies and medians with interquartile ranges (IQR)] were used to present the baseline characteristics of all patients by age, sex, ethnicity, vitamin D status, and BMI. Data normality was assessed using the Shapiro–Wilk test. Due to the skewness of all variables, which could not be satisfactorily normalised, non-parametric methods were applied. Where relevant, continuous variables were converted to categorical parameters, i.e., 25(OH)D, age, and BMI, for statistical analysis. Patients were stratified into nine age groups (0–1, 2–5, 6–10, 11–16, 17–21, 22–40, 41–60, 61–80, 80+ years) to evaluate age- and ethnicity-related differences in 25(OH)D concentrations. For vitamin D status, 25(OH)D < 25 nmol/L indicated deficiency, 25–50 nmol/L insufficiency, and >50 nmol/L replete. BMI < 18.5 denoted underweight, 18.5–24.9 normal weight, 25–29.9 overweight, and 30–34.9 obese status. Between-group comparisons were performed using the Wilcoxon signed-rank test and independent samples *t*-test. Chi-squared (χ^2^) tests were used to compare vitamin D status by ethnicity, sex, and BMI. Spearman’s correlation coefficients evaluated associations between 25(OH)D concentrations and all other variables measured. Binary logistic regression assessed the relationship between ethnicity and SARS-CoV-2 infection. All statistical analyses were conducted using IBM SPSS Statistics version 29 on a Windows 11 operating system.

## 3. Results

### 3.1. 25-Hydroxyvitamin D Concentration by Ethnicity, Age, Sex and Month of Sample Collection

Characteristics of the study population are shown in Table 1. A total of 17,619 serum 25(OH)D were processed during January–June 2020. Patients were categorised into five ethnic groups: White, Black, Asian, Mixed or Other ethnic groups. Within the Asian category, people of Indian, Pakistani, Bangladeshi, and Sri Lankan origin were the most commonly represented. The median 25(OH)D concentration was highest in White patients (Table 1). White patients were also the oldest ethnic group (Table 1).

**Figure 1:**
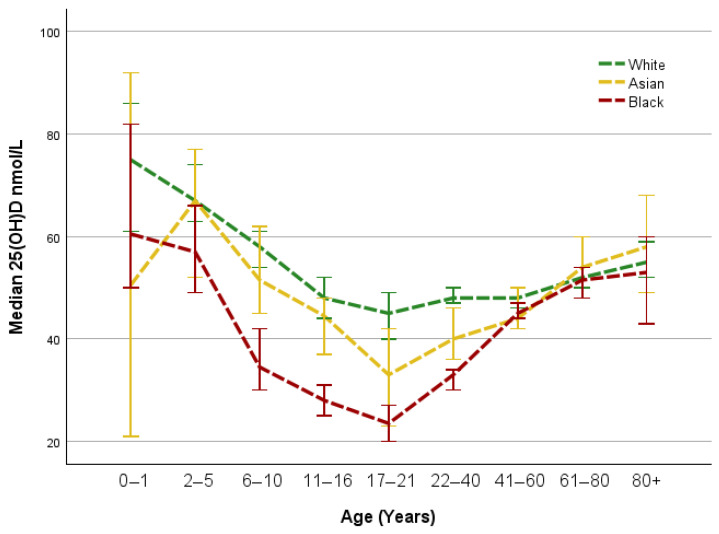
Median concentration of 25(OH)D by age and ethnicity. Group sizes: *n* = 108, 129, 201, 313, 133, 1312, 1919, 1859, 645 (White patients); *n* = 48, 80, 128, 264, 132, 736, 1340, 588, 153 (Black patients); *n* = 8, 45, 68, 90, 48, 246, 295, 178, 43 (Asian patients); for 0–1, 2–5, 6–10, 11–16, 17–21, 22–40, 41–60,61–80, 80+ years old, respectively.

Stratification by age of the three most represented ethnic groups (White, Black and Asian) showed White patients had the highest 25(OH)D concentrations across all ages ≤60 years, followed by Asian patients, with Black patients having the lowest values up to ≤60 years of age (Figure 1). The concentration of 25(OH)D was the lowest between 17 and 21 years of age for all ethnicities. 25(OH)D concentrations were similar in those >60 years of age in all ethnic groups.

**Figure 2:**
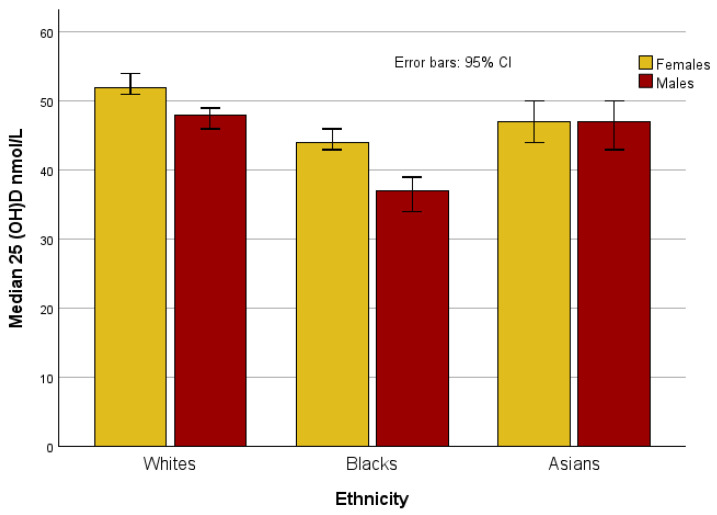
Median concentration of 25(OH)D by sex and ethnicity.

When stratified by sex, 25(OH)D concentrations were higher in Black and White females compared to males (*p* < 0.001), but there was no difference between sexes in the Asian ethnic group (*p* = 0.726) (Figure 2). Black males >40 years of age had lower 25(OH)D concentrations compared to Black females (*p* < 0.001) and Asian (*p* = 0.003) and White males (*p* < 0.001).

The median 25(OH)D were 44 nmol/L (IQR 26–65), 47 nmol/L (IQR 27–66), 52 nmol/L (IQR 33–75) for Black, Asian and White females, respectively. There was no difference in concentration between Black and Asian females (*p* = 0.139), but White females had higher concentrations than their Asian and Black counterparts (*p* < 0.001). Black males had lower 25(OH)D concentrations (median = 37 nmol/L (IQR 22–59)) (*p* < 0.001) than their White (median = 48 nmol/L (IQR 29–71)) and Asian counterparts (median = 47 nmol/L (IQR 28–68)). However, there was no difference between Asian and White males, unlike that observed for females (*p* = 0.187), Figure 2.

**Figure 3:**
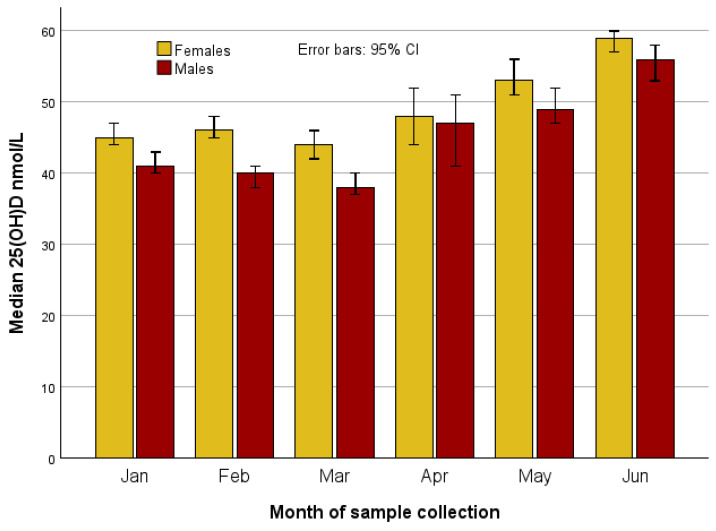
Median concentration of 25(OH)D by sex and month of sample collection.

Median 25(OH)D concentrations of the three main ethnic groups by month of sample collection, separately for females and males, showed that concentration was the highest in White females and males tested in June (Figure 3).

### 3.2. Associations of 25(OH)D with Demographic and Anthropometric Data and Selected Laboratory Results

Spearman’s correlations (correlation coefficient) of all 25(OH)D results showed positive associations with age (0.056), serum calcium (ρ = 0.181), albumin (ρ = 0.043), phosphate (ρ = 0.120), magnesium (ρ = 0.073), sodium (ρ = 0.039), folate (ρ = 0.316), total B_12_ (ρ = 0.148), mean cell volume (MCV) (ρ = 0.038) and mean corpuscular haemoglobin (MCH) (ρ = 0.045), *p* < 0.001. 25(OH)D correlated negatively with PTH (ρ = −0.245), CRP (ρ = −0.117), total bilirubin (ρ= −0.035), ALP (ρ = −0.031), RBC (ρ = −0.027), RDW (ρ = −0.086), neutrophils (ρ = −0.038), neutrophils/lymphocyte ratio (N/L) (ρ = −0.038), *p* < 0.001 and WBC count (ρ = −0.02), *p* = 0.013.

### 3.3. Prevalence of Vitamin D Deficiency and Insufficiency by Ethnicity, Sex and BMI

In Black, Asian, and White patients, 25%, 21%, and 17% were vitamin D deficient (25(OH)D < 25 nmol/L), *p* < 0.001, and 36%, 34%, and 33% were vitamin D insufficient (25–50 nmol/L), *p* < 0.001, respectively. In total, 22.4% of all males were vitamin D deficient compared with 18.5% of all females, *p* < 0.001. Vitamin D insufficiency was also more prevalent in all males (36.6%) than females (33.7%), *p* < 0.001.

**Figure 4:**
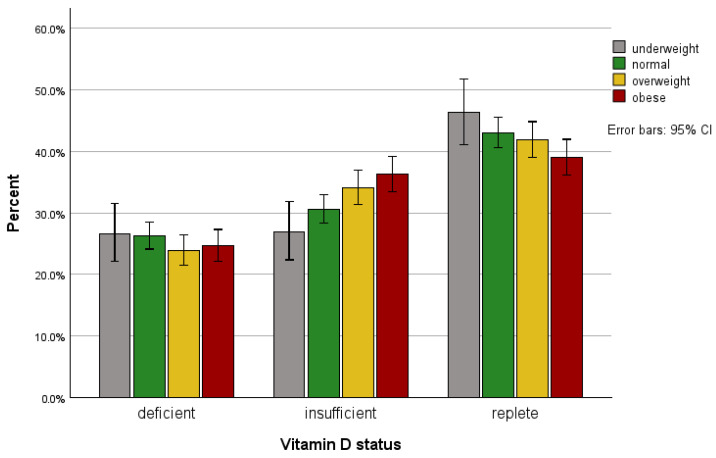
The prevalence of vitamin D deficiency (25(OH)D < 25 nmol/L), insufficiency (25(OH)D 25–50 nmol/L) and replete state (25(OH)D > 50 nmol/L) by body mass index (BMI). BMI < 18.5, 18.5–24.9, 25–29.9 and 30–34.9 defined underweight, normal, overweight and obese states, respectively.

There was no sex difference in BMI in White and Asian patients, but Black females had significantly higher BMI than males (*p* < 0.001). The prevalence of vitamin D deficiency (25(OH)D < 25 nmol/L) was higher in the underweight and patients with normal BMI compared to the overweight and obese patients (Figure 4). The prevalence of vitamin D insufficiency (25(OH)D range 25–50 nmol/L) was highest in overweight patients (33%, BMI range 25.0–29.9) and obese patients (35%, BMI range 30.0–34.9) compared to underweight patients (27%, BMI < 18.5) and those with healthy weight (31%, BMI range 18.5–24.9) (Figure 4). The prevalence of replete state (25(OH)D > 50 nmol/L) was lowest in obese patients compared to patients with BMI < 35 (Figure 4).

### 3.4. SARS-CoV-2 Positive Cases

Four hundred and eighty-five (2.8%) (median 25(OH)D 42 nmol/L (IQR 25–66)) of these 17,619 patients tested positive for SARS-CoV-2 infection. Among them, 33.4% were Black, 33.0% White, 8.9% Asian, 2.3% Chinese, 0.6% Mixed and 21.8% of Unknown ethnicity. Black patients were 1.87 times more likely to have SARS-CoV-2 infection than White patients (*p* < 0.001, 95% CI: 1.49–2.34).

Thirty-three (6.8%) patients were <17 years, 14 (2.9%) were 17–21 years, 83 (17.1%) were 22–40 years, 171 (35.3%) were 41–60 years, 118 (24.3%) were 61–80 years and 66 (13.6%) were > 80 years of age.

The prevalence of obesity (BMI > 30) was high among SARS-CoV-2-infected patients, especially for Black (28.7%) and White patients (19.8%). There was a significant difference (*p* < 0.001) in BMI between Black females and males, with 34.7% obese and 26.5% overweight females compared to 5.3% obese and 26.3% overweight males. Conversely, more White males were obese (16.7%) and overweight (28.8%) than White females (16.0% and 24.0%, respectively).

Overall, the prevalence of previously detected vitamin D deficiency was 24.1% (*n* = 117) and insufficiency 36.7% (*n* = 178) among cases that tested positive for SARS-CoV-2 infection. Additionally, 38% of those with deficiency were White (median age = 67.5 years (IQR 57.0–76.7)) and 35% Black (median age = 52.0 years (IQR 25.0–67.5)).

25(OH)D correlated negatively with markers of inflammation/cellular immunity in SARS-CoV-2 infected patients: CRP (*n* = 276, ρ = −0.183, *p =* 0.002), WBC (*n* = 457, ρ = −0.101, *p* = 0.031), neutrophils (*n* = 457, ρ = −0.118, *p* = 0.012), monocytes (*n* = 457, ρ = −0.113, *p* = 0.016) and N/L ratio (*n* = 457, ρ = −0.106, *p* = 0.023).

### 3.5. COVID-19 Positive Cases That Had Cytokines Measured

In a subset analysis of SARS-CoV-2-infected patients (*n* = 46) who were consequently referred for cytokine analysis, 25(OH)D correlated negatively with inflammation as measured by TNF-ɑ (Spearman’s ρ = −0.354, *p* = 0.016).

## 4. Discussion

### 4.1. Key Findings

This retrospective observational study provides an assessment of 25(OH)D status at the onset of the SARS-CoV-2 pandemic in a diverse pre-COVID-19 vaccine patient population living in South East London. The highest prevalence of vitamin D deficiency was found in patients of Black ethnicity, those aged 17–21 years and males. The highest prevalence of vitamin D insufficiency was also found in patients of Black ethnicity, males and those who are overweight and obese. 25(OH)D concentrations gradually decreased in children up to adulthood in all ethnicities. In contrast, 25(OH)D concentrations gradually increased from adulthood to old age in all ethnicities studied (Figure 1). Across all ethnicities, there was an association between vitamin D deficiency and insufficiency and being overweight. Several large, recently published studies have shown a negative correlation between circulating 25(OH)D and measures of overweight/obesity [4,35,36]. Moreover, it is recognised that obesity/overweight is a predictor of developing complications, the need for hospitalisation, intensive care, and mechanical ventilation and a predictor of death in SARS-CoV-2 infection [37]. Exactly what the 25(OH)D contribution in obesity is to these predictive outcome measures is unclear at present. The question remains whether 25(OH)D is a more independent risk factor than obesity, or whether there is a synergistic impact of obesity and concomitant low 25(OH)D status.

We observed no sex-related differences in BMI in White and Asian patients, except for the increased prevalence of obesity and overweight among Black females. However, several studies have demonstrated associations between BMI and vitamin D status across different ethnicities, corroborating our findings, as reported in a systematic review by Abiri et al. [4], who indicated that across all ethnicities, those with a BMI over 25 had higher levels of vitamin D inadequacy. As a fat-soluble vitamin, serum 25(OH)D concentrations might be affected by the greater volume of distribution in overweight patients [38].

Higher supplementation rates in post-menopausal women may explain sex-related differences in 25(OH)D in patients over 40 years of age. Post-menopausal women in Europe and other countries are recommended to take vitamin D supplements to aid in osteoporosis prevention and reduce the risk of fractures associated with falls and bone loss [39]. For instance, in the UK, Public Health England recommends that adults over 65 years take a daily supplement containing 10 μg (400 IU) of vitamin D to maintain sufficient levels and support bone health [40].

We also observed a negative correlation with CRP and 25(OH)D and a negative correlation between low 25(OH)D concentrations and elevated TNF-ɑ in a subset of patients with a SARS-CoV-2 infection. This is consistent with hyperinflammation as one of the mechanisms underlying the involvement of 25(OH)D in the pathology and clinical outcome of SARS-CoV-2 infection. Interestingly, obesity itself is also linked to raised pro-inflammatory biomarkers such as TNF-ɑ and IL-6 [41,42,43].

### 4.2. Vitamin D and Demographic Data

We found a higher prevalence of vitamin D deficiency in patients of Black ethnicity, which may be due to lower concentrations of dermal vitamin D production in this population [44,45]. The other reason for lower 25(OH)D in Black people may be partly related to vitamin D-binding protein (VDBP) [46]. Powe et al. suggested that lower concentrations of VDBP present in Black individuals in comparison with White people could contribute to overall lower 25(OH)D concentrations without affecting vitamin D status [47]. However, polymorphisms in the gene coding for VDBP (e.g., GC loci) are likely to influence vitamin D concentrations in people from different ethnic backgrounds [48].

Parlato et al. [49] point out that Powe et al.’s study used an assay less sensitive for the detection of the particular VDBP isotype which is more prevalent in Black populations. Black people are more likely to carry the GC1f allele, while GC1s is more prevalent in people of White ethnicity [50]. Schwartz et al. [50] found that the former is associated with lower total vitamin D concentrations; however, they note that data regarding the affinity of each isotype/polymorphism to the substrate is unclear.

Interestingly, the concentrations of 25(OH)D were the lowest in the 17–21-year-old age group. The lower 25(OH)D before age 17 could be attributed to the depletion of the vitamin’s stores in order to achieve bone maturity [51], which would also explain the subsequent rise after puberty has ended [52]. A study by Djerdjar et al. [53] in a healthy Algerian young adult population found 65.1% of the subjects to be deficient in vitamin D. Similar findings were reported by Nascimento et al. [54], who investigated the association between age, diet and vitamin D status in Brazil, reporting that most individuals between 20 and 39 years are deficient, with lower deficiency prevalence over age 40. A retrospective review by Benameur et al. [55] looking at vitamin D status before, during, and after COVID-19 lockdowns in Saudi Arabia showed a similar trend to the one we obtained in relation to patient age in this study. The lower 25(OH)D concentrations observed in children and young adults during the beginning of lockdown, when this data was collected, could also be attributed to home confinement [56], a situation less common among adults and older individuals who were more likely to have access to gardens or continue working outside.

### 4.3. Vitamin D and SARS-CoV-2 Infection

Our study results suggest that low 25(OH)D concentrations are associated with increased susceptibility to SARS-CoV-2 infection, consistent with results of other studies [57,58,59,60,61]. For example, in a large USA study (*n* = 190,000 patients from 50 states), SARS-CoV-2 positivity rates were strongly inversely correlated with the circulating 25(OH)D concentration [62]. Furthermore, in an Iranian cohort, a significant association was observed between 25(OH)D concentrations and SARS-CoV-2 infection severity [58]. Similarly, Karonova et al. [59] reported a heightened prevalence of severe SARS-CoV-2 infection cases among Russian patients with vitamin D deficiency (*p* < 0.001), particularly when 25(OH)D concentrations were below 28.5 nmol/L (*p* = 0.003). Furthermore, a genome-wide association study by Qiu et al. [60], focusing solely on individuals of European descent, identified the rs4971066 locus as potentially involved in reduced 25(OH)D concentrations and, consequently, increased susceptibility to SARS-CoV-2 infection (*r* = −0.143, *p* = 0.011) [60].

Several studies have shown beneficial clinical effects of vitamin D supplementation in SARS-CoV-2 infection, particularly in relation to lower disease severity and, in some cases, better survival outcomes and in geographically different populations. For example, Ling et al. [63], in a UK hospital setting, investigated acute inpatient SARS-CoV-2-positive patients (*n* = 542), who were serum 25(OH)D insufficient or deficient at baseline, and the effects of up to 7 weeks of treatment with 20,000 to 50,000 IU of vitamin D and found a reduced risk of mortality. In a randomised (non-double-blind) clinical study (*n* = 76) in Spain, Castillo et al. [64] demonstrated significantly reduced mortality and need for ICU treatment in hospitalised SARS-CoV-2-positive patients treated with 20,000 IU of vitamin D daily. Although the baseline levels of serum 25(OH)D were not reported by the above authors, based on other locally available population data, they suggested that their study subjects would have been deficient/insufficient. In a frail elderly hospitalised SARS-CoV-2-positive patient (*n* = 77) observational study in France, Annweiler et al. [65] found improved survival and severity outcomes with high-dose (>80,000 IU) vitamin D, although whether these patients were already serum 25(OH)D depleted was not determined. Further reviews provide more support for a beneficial role of vitamin D in SARS-CoV-2 infection. A significant association between low serum vitamin D status and increased risk of SARS-CoV-2 infection was reported in the meta-analysis (*n* = 10 studies) by Liu et al. [57], although there was evidence of publication bias. And in the systematic review by Feiner Solís et al. [66] of 11 clinical and RCT studies, they concluded that administration of a single dose of vitamin D was not an effective treatment. However, six of the eleven studies included in their study showed significant differences in some of the clinical outcome measures. In the meta-analysis, albeit a small study number (*n* = 5), undertaken by Argano et al. [67], they reported a beneficial effect of vitamin D supplementation on ICU admission and mortality. In contrast, a prospective study involving 250 healthcare workers in a New York City hospital found no significant association between serum 25(OH)D concentrations and SARS-CoV-2 infection (OR = 0.98, 95% CI: 0.80–1.20) [61], indicating that retrospective study designs may introduce significant biases.

The interplay between vitamin D status and inflammatory response has been further explored in a retrospective observational study by Saponaro et al. [68], which is consistent with our results, showing that patients with 25(OH)D concentrations ≤ 50 nmol/L exhibited significantly higher IL-6, CRP, and TNF-ɑ levels. This raises the question: could vitamin D supplementation be beneficial for high-risk groups, i.e., those with low 25(OH)D status? In a randomised controlled trial involving SARS-CoV-2-infected patients (*n* = 99), Sauša et al. [69] found an inverse relationship between daily intake of 300 μg of vitamin D_3_ and IL-6 and CRP levels in moderate to severe cases. Moreover, a case–control study indicated that vitamin D supplementation might enhance survival rates in critically ill patients [70]. Salman et al. [71] demonstrated in a randomised controlled study that administering 100 μg daily of vitamin D for two weeks reduced the median time for clinical improvement from 9 to 7 days (*p* < 0.001), as well as the median hospitalisation duration from 11 to 9 days (*p* < 0.001). However, Silva et al. [72] observed no significant difference in IL-6 and TNF-ɑ levels between patients treated with a single oral dose of 12,500 μg of vitamin D and those receiving a placebo, a finding potentially attributable to the small sample size and the nature of the intervention. Furthermore, a randomised double-blinded trial by Moghaddam et al. [73] on high-dose vitamin D supplementation daily for one month showed a reduction in ALP levels, a marker linked to high SARS-CoV-2 infection severity, in the intervention group (*p* = 0.04). Finally, in relation to SARS-CoV-2 vaccines, several studies, but not all, have reported a beneficial effect of increased serum 25(OH)D status on SARS-CoV-2 antibody titres [74,75,76,77,78]. Vitamin D status appears, therefore, to be critically important not only in relation to clinical outcomes but also SARS-CoV-2 vaccination. Thus, any large vaccination programme should be mindful of differences in vitamin D status within the population between different ethnic and other groups, e.g., age-related.

Summarising these findings, the collective data presented here, and other published data, do indicate a positive correlation between adequate vitamin D status and improved markers, such as reduced inflammatory response to SARS-CoV-2 infection. However, more research is warranted to establish serum 25(OH)D as an independent risk factor. There is therefore still a need for well-designed randomised controlled studies and meta-analyses to support clinical recommendations.

### 4.4. Generalisability

This study, which focused on a diverse urban patient population in South East London, suggests ethnicity, age and obesity/overweight influence vitamin D status and possibly its impact on SARS-CoV-2 infection. The observed link between vitamin D status and SARS-CoV-2 infection outcomes is consistent with global research, but regional differences in vitamin D deficiency prevalence and SARS-CoV-2 infection dynamics are also likely to be important.

### 4.5. Limitations

The retrospective design of our study and reliance on existing laboratory records potentially limit the accuracy of 25(OH)D concentration correlation with SARS-CoV-2 infection, as these were not measured at exactly the same points in time in most patients. Furthermore, the true incidence of SARS-CoV-2 infection within this patient cohort may have been underestimated, as not all individuals with the infection may have been tested, or tested specifically at St Thomas’ Hospital, from which the data were obtained. It also precludes definitive conclusions about causality in the vitamin D–inflammation link in SARS-CoV-2 infection. This limitation, common in vitamin D research, was discussed by Walsh et al. [79]. Other factors, such as co-morbidities prevalent in certain ethnic groups, such as chronic kidney disease and chronic liver disease, or seasonal variations due to UV light exposure might also influence vitamin D concentrations, and importantly, patient supplementation was not accounted for. Furthermore, socioeconomic factors such as overcrowded housing and economic disadvantage may also have been important determinants of infection rates as well as vitamin D status in our South East London population.

## 5. Conclusions

Vitamin D deficiency and insufficiency are prevalent in patients from South East London (UK). Age, BMI, ethnic, and sex-related differences in vitamin D status were found. Vitamin D deficiency and insufficiency were highly prevalent in people with SARS-CoV-2 infection and especially among Black people previously found to be 25(OH)D deficient. Moreover, 25(OH)D deficiency may be considered a risk factor for SARS-CoV-2 infection and associated hyperinflammation. Findings from this study in a SARS-CoV-2 pre-vaccination population are important and help inform future pandemic preparedness strategies, especially in identifying modifiable risk factors relevant to high-risk demographic groups in early outbreak phases—with particular relevance to the UK context. Monitoring vitamin D status in patient populations and addressing deficiencies will ensure adequacy of this nutrient for immune system regulation and possibly reduce infection risk.

## Figures and Tables

**Table 1:** Characteristics of the study population. Median age and 25(OH)D concentrations with interquartile ranges by ethnicity. ^1^ Population data from the National Census 2021 for Lambeth, Southwark and Lewisham—London’s boroughs served by St Thomas’ Hospital to compare with the study population.

South East London Population (%) ^1^	*n* (%) of Total	Median 25(OH)D (IQR) nmol/L	Median Age (IQR) Years	Ethnicity
52.7	6619 (37.6)	51 (31–74)	54 (34–69)	White
25.3	3469 (19.7)	42 (24–63) *	47 (29–59) *	Black
8.7	1021 (5.8)	47 (27–66) *^,^^	41 (21–57) *^,^^	Asian
7.8	357 (2.0)	42 (25–64) *	33 (15–53) *^,^^	Mixed
5.6	1323 (7.5)	44 (28–65) *^,^^^	46 (31–61) *^,^^	Other
N/A	4830 (27.4)	46 (28–68) ^NA^	41 (26–58) ^NA^	Unknown

* *p* < 0.001 compared to the White ethnic group. ^^^ *p* < 0.001 and ^^^^ *p* < 0.05 compared to the Black ethnic group. IQR, interquartile range.

## Data Availability

The original contributions presented in this study are included in the article. Further inquiries can be directed to the corresponding author.

## Abbreviations

The following abbreviations are used in this manuscript:

25(OH)D	25-hydroxyvitamin D
ACE2	Angiotensin-converting enzyme 2
BAME	Black, Asian and Minority Ethnic
BMI	Body mass index
CI	Confidence intervals
CRP	C-reactive protein
FBC	Full blood count
IQR	Interquartile range
PTH	Parathyroid hormone
TGF-β	Transforming growth factor beta
TNF-ɑ	Tumour necrosis factor alpha
UVB	Ultraviolet B
VDBP	Vitamin D-binding protein
VDR	Vitamin D receptor

## References

[1] Holt H., Talaei M., Greenig M., Zenner D., Symons J., Relton C., Young K.S., Davies M.R., Thompson K.N., Ashman J. (2022). Risk factors for developing COVID-19: A population-based longitudinal study (COVIDENCE UK). Thorax.

[2] Intensive Care National Audit Research Centre (ICNARC) (2020). ICNARC Report on COVID-19 in Critical Care.

[3] Office for National Statistics (ONS) (2022). Ethnic Group, England and Wales: Census 2021. https://www.ons.gov.uk/peoplepopulationandcommunity/culturalidentity/ethnicity/bulletins/ethnicgroupenglandandwales/census2021.

[4] Abiri B., Valizadeh M., Ahmadi A.R., Amini S., Nikoohemmat M., Abbaspour F., Hosseinpanah F. (2024). Association of vitamin D levels with anthropometric and adiposity indicators across all age groups: A systematic review of epidemiologic studies. Endocr. Connect..

[5] Ford L., Graham V., Wall A., Berg J. (2006). Vitamin D concentrations in an UK inner-city multicultural outpatient population. Ann. Clin. Biochem. Int. J. Lab. Med..

[6] NHS (2017). Vitamin D. https://www.nhs.uk/conditions/vitamins-and-minerals/vitamin-d/.

[7] SACN (2023). Vitamin D and Health Report. https://www.gov.uk/government/publications/sacn-vitamin-d-and-health-report.

[8] Sutherland J.P., Zhou A., Leach M.J., Hyppönen E. (2021). Differences and determinants of vitamin D deficiency among UK biobank participants: A cross-ethnic and socioeconomic study. Clin. Nutr..

[9] Wimalawansa S.J. (2023). Infections and Autoimmunity—The Immune System and Vitamin D: A Systematic Review. Nutrients.

[10] Hanel A., Bendik I., Carlberg C. (2021). Transcriptome-Wide Profile of 25-Hydroxyvitamin D_3_ in Primary Immune Cells from Human Peripheral Blood. Nutrients.

[11] Voltan G., Cannito M., Ferrarese M., Ceccato F., Camozzi V. (2023). Vitamin D: An Overview of Gene Regulation, Ranging from Metabolism to Genomic Effects. Genes.

[12] Carlberg C. (2022). Vitamin D and Its Target Genes. Nutrients.

[13] Bikle D.D. (2008). Vitamin D and the immune system: Role in protection against bacterial infection. Curr. Opin. Nephrol. Hypertens..

[14] Lin R., White J.H. (2004). The pleiotropic actions of vitamin D. BioEssays News Rev. Mol. Cell. Dev. Biol..

[15] Bishop E., Ismailova A., Dimeloe S., Hewison M., White J.H. (2021). Vitamin D and Immune Regulation: Antibacterial, Antiviral, Anti-Inflammatory. JBMR Plus.

[16] Tripathi A.K., Mishra S.K. (2023). A review article on neuroprotective, immunomodulatory, and anti-inflammatory role of vitamin-D3 in elderly COVID-19 patients. Egypt. J. Neurol. Psychiatry Neurosurg..

[17] Hansdottir S., Monick M.M., Lovan N., Powers L., Gerke A., Hunninghake G.W. (2010). Vitamin D Decreases Respiratory Syncytial Virus Induction of NF-κB–Linked Chemokines and Cytokines in Airway Epithelium While Maintaining the Antiviral State. J. Immunol..

[18] Alabdullatif W., Almnaizel A., Alhijji A., Alshathri A., Albarrag A., Bindayel I. (2023). Correlation of Plasma 25(OH)D_3_ and Vitamin D Binding Protein Levels with COVID-19 Severity and Outcome in Hospitalized Patients. Nutrients.

[19] Bayraktar N., Turan H., Bayraktar M., Ozturk A., Erdoğdu H. (2022). Analysis of serum cytokine and protective vitamin D levels in severe cases of COVID-19. J. Med. Virol..

[20] Larriba M.J., González-Sancho J.M., Bonilla F., Muñoz A. (2014). Interaction of vitamin D with membrane-based signaling pathways. Front. Physiol..

[21] Hart P.H., Gorman S., Finlay-Jones J.J. (2011). Modulation of the immune system by UV radiation: More than just the effects of vitamin D?. Nat. Rev. Immunol..

[22] Ma J.G., Wu G.J., Xiao H.L., Xiao Y.M., Zha L. (2021). Vitamin D has an effect on airway inflammation and Th17/Treg balance in asthmatic mice. Kaohsiung J. Med. Sci..

[23] Zhou Q., Qin S., Zhang J., Zhon L., Pen Z., Xing T. (2017). 1,25(OH)_2_D_3_ induces regulatory T cell differentiation by influencing the VDR/PLC-γ1/TGF-β1/pathway. Mol. Immunol..

[24] Abo-Zaid M.A., Hamdi H.A., Elashmawy N.F. (2023). Vitamin D and Immunity: A comprehensive review of its impact on autoimmunity, allergy suppression, antimicrobial defense, and cancer inhibition. Egypt. J. Immunol..

[25] Shawky N.M., EL-Antouny N.G., Hassaan N.K., Abdullah A.A. (2022). Emerging Relationship between Vitamin D and LL-37 in the Immune System’s Response to Infection and Their Possible Role in Combating Sepsis. Egypt. J. Hosp. Med..

[26] Gotelli E., Soldano S., Hysa E., Casabella A., Cere A., Pizzorni C., Paolino S., Sulli A., Smith V., Cutolo M. (2023). Understanding the Immune-Endocrine Effects of Vitamin D in SARS-CoV-2 Infection: A Role in Protecting against Neurodamage. Neuroimmunomodulation.

[27] Wang T.T., Nestel F.P., Bourdeau V., Nagai Y., Wang Q., Liao J., Tavera-Mendoza L., Lin R., Hanrahan J.W., Mader S. (2004). Cutting Edge: 1,25-Dihydroxyvitamin D3 Is a Direct Inducer of Antimicrobial Peptide Gene Expression. J. Immunol..

[28] Coperchini F., Greco A., Denegri M., Ripepi F.A., Grillini B., Bertini J., Calì B., Villani L., Magri F., Croce L. (2022). Vitamin D and interferon-γ co-operate to increase the ACE-2 receptor expression in primary cultures of human thyroid cells. J. Endocrinol. Investig..

[29] Yalcin H.C., Sukumaran V., Al-Ruweidi M.K.A., Shurbaji S. (2021). Do Changes in ACE-2 Expression Affect SARS-CoV-2 Virulence and Related Complications: A Closer Look into Membrane-Bound and Soluble Forms. Int. J. Mol. Sci..

[30] Hawerkamp H.C., Dyer A.H., Patil N.D., McElheron M., O’Dowd N., O’Doherty L., Mhaonaigh A.U., George A.M., O’Halloran A.M., Reddy C. (2023). Characterisation of the pro-inflammatory cytokine signature in severe COVID-19. Front. Immunol..

[31] Attwood L.O., Francis M.J., Hamblin J., Korman T.M., Druce J., Graham M. (2020). Clinical evaluation of AusDiagnostics SARS-CoV-2 multiplex tandem PCR assay. J. Clin. Virol..

[32] Stevens B.A., Hogan C.A., Mfuh K.O., Khan G., Sahoo M.K., Huang C., Garamani N., Zehnder J., Kurzer J., Pinsky B.A. (2021). Combined SARS-CoV-2 nucleic acid amplification testing and respiratory virus panel RT-PCR on the Hologic Panther Fusion system. J. Clin. Virol..

[33] Prince H.E., Givens T.S., Lapé-Nixon M., Clarke N.J., Schwab D.A., Batterman H.J., Jones R.S., Meyer W.A., Kapoor H., Rowland C.M. (2020). Detection of SARS-CoV-2 IgG Targeting Nucleocapsid or Spike Protein by Four High-Throughput Immunoassays Authorized for Emergency Use. J. Clin. Microbiol..

[34] Mazaheri T., Ranasinghe R., Al-Hasani W., Luxton J., Kearney J., Manning A., Dimitriadis G.K., Mare T., Vincent R.P. (2022). A cytokine panel and procalcitonin in COVID-19, a comparison between intensive care and non-intensive care patients. PLoS ONE.

[35] Qiu L., Ren Y., Li J., Li M., Li W., Qin L., Zhang J., Gao F. (2024). The correlation of obesity status with serum 25-hydroxyvitamin D in US Asian adults: NHANES 2011–2018. PLoS ONE.

[36] Haghighat N., Sohrabi Z., Bagheri R., Akbarzadeh M., Esmaeilnezhad Z., Ashtary-Larky D., Barati-Boldaji R., Zare M., Amini M., Hosseini S.V. (2023). A Systematic Review and Meta-Analysis of Vitamin D Status of Patients with Severe Obesity in Various Regions Worldwide. Obes. Facts.

[37] World Obesity Federation (2021). COVID-19 and Obesity: The 2021 Atlas. https://www.worldobesity.org/resources/resource-library/covid-19-and-obesity-the-2021-atlas.

[38] Alzohily B., AlMenhali A., Gariballa S., Munawar N., Yasin J., Shah I. (2024). Unraveling the complex interplay between obesity and vitamin D metabolism. Sci. Rep..

[39] Rentzeperi E., Pegiou S., Tsakiridis I., Kalogiannidis I., Kourtis A., Mamopoulos A., Athanasiadis A., Dagklis T. (2023). Diagnosis and Management of Osteoporosis: A Comprehensive Review of Guidelines. Obstet. Gynecol. Surv..

[40] Public Health England (PHE), National Institute for Health and Care Excellence (NICE) (2020). Statement from PHE and NICE on vitamin D supplementation during winter. https://www.gov.uk/government/publications/vitamin-d-supplementation-during-winter-phe-and-nice-statement/statement-from-phe-and-nice-on-vitamin-d-supplementation-during-winter.

[41] Sethi J.K., Hotamisligil G.S. (2021). Metabolic Messengers: Tumour necrosis factor. Nat. Metab..

[42] Kawai T., Autieri M.V., Scalia R. (2021). Adipose tissue inflammation and metabolic dysfunction in obesity. Am. J. Physiol.-Cell Physiol..

[43] Al Khathlan N. (2023). Association of inflammatory cytokines with obesity and pulmonary function testing. PLoS ONE.

[44] Loomis W.F. (1967). Skin-Pigment Regulation of Vitamin-D Biosynthesis in Man. Science.

[45] Dawson-Hughes B. (2004). Racial/ethnic considerations in making recommendations for vitamin D for adult and elderly men and women2. Am. J. Clin. Nutr..

[46] Bikle D.D., Schwartz J. (2019). Vitamin D Binding Protein, Total and Free Vitamin D Levels in Different Physiological and Pathophysiological Conditions. Front. Endocrinol..

[47] Powe C.E., Evans M.K., Wenger J., Zonderman A.B., Berg A.H., Nalls M., Tamez H., Zhang D., Bhan I., Karumanchi S.A. (2013). Vitamin D-binding protein and vitamin D status of black Americans and white Americans. N. Engl. J. Med..

[48] Khan A., Jafri L., Siddiqui A., Naureen G., Morris H., Moatter T. (2019). Polymorphisms in the GC Gene for Vitamin D Binding Protein and Their Association with Vitamin D and Bone Mass in Young Adults. J. Coll. Physicians Surg. Pak..

[49] Parlato L.A., Welch R., Ong I.M., Long J., Cai Q., Steinwandel M.D., Blot W.J., Zheng W., Andersen S.W. (2023). Genome-wide association study (GWAS) of circulating vitamin D outcomes among individuals of African ancestry. Am. J. Clin. Nutr..

[50] Schwartz J.B., Gallagher J.C., Jorde R., Berg V., Walsh J., Eastell R., Evans A.L., Bowles S., Naylor K.E., Jones K.S. (2018). Determination of Free 25(OH)D Concentrations and Their Relationships to Total 25(OH)D in Multiple Clinical Populations. J. Clin. Endocrinol. Metab..

[51] Tylavsky F.A., Ryder K.M., Li R., Park V., Womack C., Norwood J., Carbone L.D., Cheng S. (2007). Preliminary Findings: 25(OH)D Levels and PTH Are Indicators of Rapid Bone Accrual in Pubertal Children. J. Am. Coll. Nutr..

[52] Karacan M., Usta A., Biçer S., Baktir G., Gündogan G., Usta C., Akinci G. (2020). Serum vitamin D levels in healthy urban population at reproductive age: Effects of age, gender and season. Cent. Eur. J. Public Health.

[53] Djerdjar L., Ramdane S., Oussadou L. (2022). Epidemiology of hypovitaminosis D among apparently healthy young adults in Algeria. Rev. Med. Brux..

[54] Nascimento L.M., de Carvalho Lavôr L.C., de Lima Sousa P.V., Luzia L.A., Viola P.C.D.A.F., de Azevedo Paiva A., de Carvalho Rondó P.H., Frota K.D.M.G. (2023). Consumption of ultra-processed products is associated with vitamin D deficiency in Brazilian adults and elderly. Br. J. Nutr..

[55] Benameur T., Kaliyadan F., Saidi N., Porro C. (2023). A Retrospective Chart Review Evaluating Changes in 25-Hydroxyvitamin D Levels among Patients Attending the University Healthcare Centre during the COVID-19 Pandemic. Nutrients.

[56] Rustecka A., Maret J., Drab A., Leszczyńska M., Tomaszewska A., Lipińska-Opałka A., Będzichowska A., Kalicki B., Kubiak J.Z. (2021). The Impact of COVID-19 Pandemic during 2020–2021 on the Vitamin D Serum Levels in the Paediatric Population in Warsaw, Poland. Nutrients.

[57] Liu N., Sun J., Wang X., Zhang T., Zhao M., Li H. (2021). Low vitamin D status is associated with coronavirus disease 2019 outcomes: A systematic review and meta-analysis. Int. J. Infect. Dis. IJID Off. Publ. Int. Soc. Infect. Dis..

[58] Fatemi A., Ardehali S.H., Eslamian G., Noormohammadi M., Malek S. (2021). Association of vitamin D deficiency with COVID-19 severity and mortality in Iranian people: A prospective observational study. Acute Crit. Care.

[59] Karonova T.L., Kudryavtsev I.V., Golovatyuk K.A., Aquino A.D., Kalinina O.V., Chernikova A.T., Zaikova E.K., Lebedev D.A., Bykova E.S., Golovkin A.S. (2022). Vitamin D Status and Immune Response in Hospitalized Patients with Moderate and Severe COVID-19. Pharmaceuticals.

[60] Qiu S., Zheng K., Hu Y., Liu G. (2023). Genetic correlation, causal relationship, and shared loci between vitamin D and COVID-19: A genome-wide cross-trait analysis. J. Med. Virol..

[61] Liu Y., Clare S., D’Erasmo G., Heilbronner A., Dash A., Krez A., Zaworski C., Haseltine K., Serota A., Miller A. (2023). Vitamin D and SARS-CoV-2 Infection: SERVE Study (SARS-CoV-2 Exposure and the Role of Vitamin D among Hospital Employees). J. Nutr..

[62] Kaufman H.W., Niles J.K., Kroll M.H., Bi C., Holick M.F. (2020). SARS-CoV-2 positivity rates associated with circulating 25-hydroxyvitamin D levels. PLoS ONE.

[63] Ling S.F., Broad E., Murphy R., Pappachan J.M., Pardesi-Newton S., Kong M.F., Jude E.B. (2020). High-Dose Cholecalciferol Booster Therapy is Associated with a Reduced Risk of Mortality in Patients with COVID-19: A Cross-Sectional Multi-Centre Observational Study. Nutrients.

[64] Castillo M.E., Costa L.M.E., Barrios J.M.V., Díaz J.F.A., Miranda J.L., Bouillon R., Gomez J.M.Q. (2020). Effect of calcifediol treatment and best available therapy versus best available therapy on intensive care unit admission and mortality among patients hospitalized for COVID-19: A pilot randomized clinical study. J. Steroid Biochem. Mol. Biol..

[65] Annweiler G., Corvaisier M., Gautier J., Dubée V., Legrand E., Sacco G., Annweiler C. (2020). Vitamin D Supplementation Associated to Better Survival in Hospitalized Frail Elderly COVID-19 Patients: The GERIA-COVID Quasi-Experimental Study. Nutrients.

[66] Feiner Solís Á., Avedillo Salas A., Luesma Bartolomé M.J., Santander Ballestín S. (2022). The Effects of Vitamin D Supplementation in COVID-19 Patients: A Systematic Review. Int. J. Mol. Sci..

[67] Argano C., Mallaci Bocchio R., Natoli G., Scibetta S., Lo Monaco M., Corrao S. (2023). Protective Effect of Vitamin D Supplementation on COVID-19-Related Intensive Care Hospitalization and Mortality: Definitive Evidence from Meta-Analysis and Trial Sequential Analysis. Pharmaceuticals.

[68] Saponaro F., Franzini M., Okoye C., Antognoli R., Campi B., Scalese M., Neri T., Carrozzi L., Monzani F., Zucchi R. (2022). Is There a Crucial Link Between Vitamin D Status and Inflammatory Response in Patients With COVID-19?. Front. Immunol..

[69] Sauša S., Kistkins S., Krūzmane L., Kalniņa D., Jurģe B., Ivanova K., Svikle Z., Frīdvalde A., Roškova V., Zariņa R.E. (2023). Impact of Vitamin D Therapy on C-Reactive Protein, Ferritin, and IL-6 Levels in Hospitalised Covid-19 Patients. Proc. Latv. Acad. Sci. Sect. B Nat. Exact Appl. Sci..

[70] Gholamalizadeh M., Rabbani F., Ahmadzadeh M., Hajipour A., Musavi H., Mobarakeh K.A., Salimi Z., Bahar B., Mahmoodi Z., Gholami S. (2023). The association between vitamin D intake with inflammatory and biochemical indices and mortality in critically ill patients with COVID-19: A case-control study. Immun. Inflamm. Dis..

[71] Salman M., Zaman S., Aymun U., Khawar S., Khan I., Karim A. (2023). Role of Vitamin-D Supplementation in COVID-19 Patients. Biol. Clin. Sci. Res. J..

[72] Silva M.G., Inserra F., Mariani J., Antonietti L., Nuñez M., Tajer C., Ferder L., Inserra P.I., Ross F., Cunto M.S. (2023). Mechanistic approaching study in COVID-19 patients treated with high doses of vitamin D. Explor. Med..

[73] Moghaddam R.R., Khorasanchi Z., Noor A.R., Moghadam M.S.F., Esfahani A.J., Alyakobi A.K.M., Alboresha M.L., Sharifan P., Bahari A., Rezvani R. (2023). High-dose vitamin D supplementation is related to an improvement in serum alkaline phosphatase in COVID-19 patients; a randomized double-blinded clinical trial. J. Health Popul. Nutr..

[74] Piec I., Cook L., Dervisevic S., Fraser W.D., Ruetten S., Berman M., English E., John W.G. (2022). Age and vitamin D affect the magnitude of the antibody response to the first dose of the SARS-CoV-2 BNT162b2 vaccine. Curr. Res. Transl. Med..

[75] Parthymou A., Habeos E.E., Habeos G.I., Deligakis A., Livieratos E., Marangos M., Chartoumpekis D.V. (2022). Factors associated with anti-SARS-CoV-2 antibody titres 3 months post-vaccination with the second dose of BNT162b2 vaccine: A longitudinal observational cohort study in western Greece. BMJ Open.

[76] Abu Fanne R., Lidawi G., Maraga E., Moed M., Roguin A., Meisel S.R. (2022). Correlation between Baseline 25(OH) Vitamin D Levels and Both Humoral Immunity and Breakthrough Infection Post-COVID-19 Vaccination. Vaccines.

[77] di Filippo L., Frara S., Terenzi U., Nannipieri F., Locatelli M., Ciceri F., Giustina A. (2023). Lack of vitamin D predicts impaired long-term immune response to COVID-19 vaccination. Endocrine.

[78] Cesur F., Atasever Z., Özoran Y. (2023). Impact of vitamin D3 supplementation on COVID-19 vaccine response and immunoglobulin G antibodies in deficient women: A randomized controlled trial. Vaccine.

[79] Walsh J.B., McCartney D.M., Laird É., McCarroll K., Byrne D.G., Healy M., O’Shea P.M., Kenny R.A., Faul J.L. (2022). Understanding a Low Vitamin D State in the Context of COVID-19. Front. Pharmacol..

